# The impact of different care modes on the rehabilitation efficacy of acute ischemic stroke patients

**DOI:** 10.1186/s12883-026-04789-6

**Published:** 2026-04-06

**Authors:** Wenjuan Song, Shengyong Wu, Lei Shi, Xiaowei Luan, Jialiang Song, Jintao Lai, Cheng Wu, Fanfu Fang

**Affiliations:** 1 Department of Rehabilitation, Changhai Hospital, Naval Medical University, Shanghai, 200433 China; 2 Department of statistics, Naval Medical University, Shanghai, 200433 China; 3 Department of Rehabilitation, Shanghai 411 Hospital, China RongTong Medical Healthcare Group Co.Ltd, Shanghai, 200081 China

**Keywords:** Stroke, Caregiving, Family, Rehabilitation, Orderly

## Abstract

**Objective:**

To explore the differences of the risk of complications, functional rehabilitation, and hospitalization expenses between the ischemic stroke patients cared by their families or orderlies.

**Methods:**

This was a retrospective cohort study. We collected a total of 443 patients with ischemic stroke. According to the different types of nursing staff, they were divided into family caregiving group and orderly caregiving group. Propensity Score Weighting (PSW) method was used to balance the baseline; The weighted logistic regression model was used to analyze the risk of pulmonary, urinary tract infection, diarrhea, falls, and pressure ulcer in different groups of patients. The functional rehabilitation indicators, including the National Institutes of Health Stroke Scale (NIHSS) score, Barthel score, muscle strength grade and hospitalization cost before discharge, were compared between the two groups by means of independent samples *t* test or Wilcoxon rank sum test, depending on the distribution characteristics of the data. *P* value less than 0.05 was considered statistically significant.

**Results:**

①There were no significant differences in the incidence of pulmonary, urinary tract infection, diarrhea and fall between the two groups. The family caregiving group had a lower risk of developing pressure ulcer. ②The proportion of patients with upper limb muscle strength of grade 3 or above in the family caregiving group (90.60%) was higher than that in the orderly caregiving group (82.26%). ③The hospitalization expenses of the family caregiving group, and the average daily hospitalization expenses were higher than those of the orderly caregiving group, respectively.

**Conclusion:**

Family caregiving is a protective factor for pressure ulcer in ischemic stroke patients.The patients in the family caregiving group had better upper limb muscle strength.

## Introduction

Stroke is the second leading cause of death and the third major cause of disability in the world [1]. Complications in stroke patients include infection, falls, stroke recurrence and progression, pain, thromboembolism, etc [2]. These complications can delay or hinder the recovery process, lead to greater functional impairment, and affect prognosis [3].The person with the most contact time and the closest contact with the patient is the patient’s caregivers. The quality of caregivers’ supports plays an important role in the rehabilitation of stroke patients.

Currently, the field of hospital nursing in China presents distinct developmental characteristics: there exists a shortage in the total nursing workforce; meanwhile, the personnel structure shows a trend of rising educational attainment and younger age profile. In clinical practice, nurses are primarily responsible for ward rounds and professional nursing care, while the non-technical daily living assistance for patients has been gradually undertaken by hospital orderlies. Typically, hospital orderlies are stationed in medical institutions through a third-party dispatch mechanism, where the dispatch agencies sign cooperation agreements with hospitals. At present, with the arrival of an aging society in China, many patients from one-child family and empty-nest elderly people have limited family member, they need orderly to provide bedside rehabilitation assistance [4]. What are the differences between the two types of accompanying modes, family members and orderly, will they affect the patient’s recovery?

During the COVID-19 pandemic, different countries and regions have also implemented different visit restrictions [5]. Our hospital had a restricted visitation and bedside care policy in the inpatient ward. Only one regular caregiver was allowed to accompany and the previous dual support model of “family relative + orderlies” was changed to a single care model of one family relative or one orderly.The research group carried out a standardized retrospective cohort study to analyze the differences in complications, functional rehabilitation and hospitalization expenses of ischemic stroke patients under the two escort modes.

## Materials and methods

### Research objects

A total of 855 ischemic stroke patients admitted to the Cerebrovascular Center of Changhai Hospital from April 1, 2020 to December 31, 2021 were collected and screened, 443 among them met the inclusion criteria. According to the nursing records, the qualified patients during hospitalization were divided into the patients cared by their own family (family caregiving group) and those cared by orderly (orderly caregiving group). We collected the patients’ general conditions and disease information, such as gender, age, Body Mass Index (BMI), comorbidities, smoking history, excessive drinking, feeding methods, urethral catheterization, treatment approach, cause of disease, infarct foci size, clinical type, and the scale scores and inspection indicators at admission and discharge.

### Patient enrollment

#### Inclusion criteria

Patients should meet the diagnostic criteria for ischemic stroke, with age ≥ 20 years, Glasgow Coma Scale (GCS) > 12 points [6], and National Institutes of Health Stroke Scale (NIHSS) ≤ 15 points [7]. The course of disease is within 1 to 7 days. The hospitalization time is 4 to 21 days. The patients should receive either conservative medication or thrombolytic therapy. During hospitalization, they should be accompanied by family or orderly who were not replaced, and they have not been transferred to other departments or the intensive care unit.

#### Exclusion criteria

Patients should not be complicated with cerebral hemorrhage, infectious diseases, organ failure, malignant tumor, gastrointestinal bleeding, intracranial tumor, traumatic brain injury, or arteriovenous malformation. Patients should not have the history of stroke or major trauma in the past 3 months. They should not have neurological or musculoskeletal diseases affecting functional recovery, or severe mental illness. They should not have pre-hospital infection, pressure ulcers, visuospatial defects, ataxia, Parkinson’s disease and other diseases are not allowed. Those who lack baseline data are also be excluded.

### Outcome indicators

The primary outcome was the risk of hospital-based medical complications, including pulmonary [8], urinary tract infection [8], diarrhea [8], falls [9], pressure ulcer [10] and other complications. The secondary outcomes were NIHSS score before discharge, Barthel score [11] before discharge, muscle strengths of the upper and lower limbs before discharge, functional rehabilitation outcomes such as upper and lower limb muscle strength before discharge and hospitalization costs. Outcome indicators are collected through discharge summaries, progress notes, nursing records, and billing statements. The incidence of various complications is calculated as the proportion of patients who experience a specific complication relative to the total number of patients included in the study during the same period.

### Statistical analysis

The measurement data conforming to the normal distribution were expressed as $\stackrel{-}{\boldsymbol{x}}$±s, the measurement data with non-normal distribution were expressed as M(Q25-Q75), and the enumeration data were expressed as percentage (%). The comparison of measurement data between groups was performed using independent samples *t* test or Mann-Whitney U test, and the comparison of enumeration data was performed using χ2 test or Fisher’s exact test.

The Propensity Score Weighting (PSW) method was used to balance the differences in covariates between groups for complications. The weighted effect was evaluated by Standardized Mean Difference (SMD) between the two groups of patients at baseline, with SMD < 0.1 as the balance index. A weighted logistic regression model was used to analyze the incidence of complications in different groups of patients.The results were subjected to sensitivity analysis using multivariate logistic regression.

For secondary outcome indicators, focusing on the weighted study population, independent samples *t* test was used for measurement data that conformed to normal distribution, Wilcoxon rank-sum test was used for measurement data that did not conform to normal distribution, and χ2 test or Fisher exact test was used for enumeration data. The categorical data were analyzed using the Wilcoxon rank sum test.

A *P* value less than 0.05 was considered statistically significant. Statistical analysis was performed using SAS version 9.4 (SAS Institute Inc.) and R version 4.0.4 (R Foundation for Statistical Computing).

## Results

A total of 443 subjects were included in this study, of which 84 (18.96%) were in the orderly caregiving group and 359 (81.04%) were in the family caregiving group. The proportion of family caregiving group was higher than that of the orderly caregiving group. The patients under the age of 65 had the highest proportion of family caregiving. The numbers of orderly and family caregiving for patients with no spouse were close. There was no significant association between child status and types of caregiving. The other baseline data of the patients were shown in the (Table 1).

**Table 1:** Basic information of patients

Item		Before PSW					After PSW				
	Classification	Full Cohorts	orderly	family	*P* value	Statistic	Full Cohorts	orderly	family	*P* value	SMD
Gender	male	325(73.36)	59(70.24)	266(74.09)	0.4716	0.52	622(69.94)	299(66.82)	323(73.09)	0.0414	0.06
	female	118(26.64)	25(29.76)	93(25.91)			268(30.06)	149(33.18)	119(26.91)		
Age	<65	198(44.70)	25(29.76)	173(48.19)	0.0006	3.42	403(45.33)	206(46.06)	197(44.59)	0.9744	0.01
	65–75	176(39.73)	38(45.24)	138(38.44)			346(38.83)	169(37.76)	177(39.91)		0.02
	>75	69(15.58)	21(25.00)	48(13.37)			141(15.84)	72(16.18)	69(15.50)		0.01
Marriage	no spouse	12(2.71)	5(5.95)	7(1.95)	0.0419	4.14	22(2.47)	11(2.43)	11(2.51)	0.9364	< 0.01
	spouse	431(97.29)	79(94.05)	352(98.05)			868(97.53)	437(97.57)	431(97.49)		
Child	childless	20(4.51)	6(7.14)	14(3.90)	0.1975	1.66	41(4.60)	22(4.84)	19(4.35)	0.7235	< 0.01
	having one or more children	423(95.49)	78(92.86)	345(96.10)			849(95.40)	426(95.16)	423(95.65)		
BMI	<28 kg/㎡	366(82.62)	68(80.95)	298(83.01)	0.6544	0.20	727(81.74)	362(80.85)	366(82.65)	0.4883	0.02
	≥ 28 kg/㎡	77(17.38)	16(19.05)	61(16.99)			162(18.26)	86(19.15)	77(17.35)		
History of cerebral infarction	no	399(90.07)	74(88.10)	325(90.53)	0.5019	0.45	797(89.55)	398(88.82)	399(90.29)	0.4736	0.01
	yes	44(9.93)	10(11.90)	34(9.47)			93(10.45)	50(11.18)	43(9.71)		
Hypertension	no	89(20.09)	19(22.62)	70(19.50)	0.5205	0.41	189(21.19)	100(22.24)	89(20.12)	0.4385	0.02
	yes	354(79.91)	65(77.38)	289(80.50)			701(78.81)	348(77.76)	353(79.88)		
Heart disease	no	352(79.46)	63(75.00)	289(80.50)	0.2612	1.26	708(79.56)	355(79.23)	353(79.90)	0.8057	0.01
	yes	91(20.54)	21(25.00)	70(19.50)			182(20.44)	93(20.77)	89(20.10)		
Diabetes	no	243(54.85)	46(54.76)	197(54.87)	0.9851	0.00	511(57.41)	268(59.96)	242(54.82)	0.1208	0.05
	yes	200(45.15)	38(45.24)	162(45.13)			379(42.59)	179(40.04)	200(45.18)		
Hyperlipidemia	no	388(87.58)	70(83.33)	318(88.58)	0.1893	1.72	786(88.37)	399(89.09)	388(87.63)	0.4953	0.01
	yes	55(12.42)	14(16.67)	41(11.42)			104(11.63)	49(10.91)	55(12.37)		
Other illnesses	no	373(84.20)	75(89.29)	298(83.01)	0.1556	2.02	742(83.38)	370(82.61)	372(84.15)	0.5381	0.02
	yes	70(15.80)	9(10.71)	61(16.99)			148(16.62)	78(17.39)	70(15.85)		
Smoking history	no	261(58.92)	54(64.29)	207(57.66)	0.2665	1.23	535(60.09)	273(61.08)	261(59.08)	0.5425	0.02
	yes	182(41.08)	30(35.71)	152(42.34)			355(39.91)	174(38.92)	181(40.92)		
Excessive drinking	no	348(78.56)	73(86.90)	275(76.60)	0.0383	4.29	721(80.97)	372(83.04)	349(78.87)	0.1129	0.04
	yes	95(21.44)	11(13.10)	84(23.40)			169(19.03)	76(16.96)	93(21.13)		
Cause of disease	Large atherosclerotic type	207(46.73)	41(48.81)	166(46.24)	0.6656	2.38	435(48.87)	225(50.19)	210(47.55)	0.8953	0.03
	Cardioembolic type	23(5.19)	5(5.95)	18(5.01)			44(4.93)	22(4.86)	22(5.00)		< 0.01
	Arteriolar occlusive type	161(36.34)	27(32.14)	134(37.33)			305(34.31)	146(32.71)	159(35.92)		0.03
	Other causes	12(2.71)	4(4.76)	8(2.23)			23(2.55)	12(2.60)	11(2.50)		< 0.01
	Type of unknown cause	40(9.03)	7(8.33)	33(9.19)			83(9.33)	43(9.63)	40(9.02)		0.01
Infarct foci size	<1.5 cm	241(54.40)	43(51.19)	198(55.15)	0.4264	0.80	461(51.82)	222(49.69)	239(53.97)	0.0539	0.04
	1.6~ 3cm	125(28.22)	24(28.57)	101(28.13)			260(29.20)	133(29.65)	127(28.74)		0.01
	3.1~5cm	48(10.84)	10(11.90)	38(10.58)			91(10.19)	43(9.62)	48(10.76)		0.01
	>5 cm	29(6.55)	7(8.33)	22(6.13)			78(8.80)	49(11.04)	29(6.53)		0.05
Clinical type	complete anterior circulation infarction	12(2.71)	3(3.57)	9(2.51)	0.3754	3.11	20(2.27)	9(1.91)	12(2.65)	0.1571	0.01
	partial anterior circulation infarction	231(52.14)	49(58.33)	182(50.70)			433(48.63)	203(45.28)	230(52.02)		0.07
	posterior circulation infarction	146(32.96)	21(25.00)	125(34.82)			323(36.27)	176(39.27)	147(33.24)		0.06
	lacunar infarction	54(12.19)	11(13.10)	43(11.98)			114(12.82)	61(13.54)	54(12.10)		0.01
Treatment approach	non-thrombolytic therapy	409(92.33)	74(88.10)	335(93.31)	0.1057	2.62	831(93.32)	421(94.06)	410(92.57)	0.3726	0.01
	thrombolysis	34(7.67)	10(11.90)	24(6.69)			59(6.68)	27(5.94)	33(7.43)		
Feeding methods	Transoral	161(36.34)	28(33.33)	133(37.05)	0.5241	0.41	302(33.99)	141(31.61)	161(36.39)	0.1323	0.05
	Nasal feeding	282(63.66)	56(66.67)	226(62.95)			588(66.01)	306(68.39)	281(63.61)		
Urethral catheter	no	437(98.65)	81(96.43)	356(99.16)	0.0508	3.81	878(98.69)	442(98.70)	437(98.68)	0.9771	< 0.01
	yes	6(1.35)	3(3.57)	3(0.84)			12(1.31)	6(1.30)	6(1.32)		
Albumin index	≥ 35	417(94.13)	78(92.86)	339(94.43)	0.5811	0.30	834(93.66)	418(93.39)	416(93.93)	0.7405	0.01
	<35	26(5.87)	6(7.14)	20(5.57)			56(6.34)	30(6.61)	27(6.07)		
NIHSS score (on admission)	0 ~ 5	349(78.78)	62(73.81)	287(79.94)	0.2157	1.53	673(75.64)	325(72.58)	348(78.73)	0.0324	0.06
	6 ~ 15	94(21.22)	22(26.19)	72(20.06)			217(24.36)	123(27.42)	94(21.27)		
Barthel score (on admission)	≤ 40	63(14.22)	17(20.24)	46(12.81)	0.0704	-1.81	140(15.68)	76(16.91)	64(14.44)	0.1533	0.02
	41 ~ 60	57(12.87)	12(14.29)	45(12.53)			116(13.07)	60(13.38)	56(12.76)		0.01
	>60	323(72.91)	55(65.48)	268(74.65)			634(71.25)	312(69.71)	322(72.80)		0.03
Muscle strength of affected upper limb (on admission)	0–2	61(13.77)	13(15.48)	48(13.37)	0.6141	0.25	145(16.35)	85(18.99)	61(13.68)	0.0322	0.05
	3–5	382(86.23)	71(84.52)	311(86.63)			744(83.65)	363(81.01)	382(86.32)		
Muscle strength of affected lower limb (on admission)	0–2	34(7.67)	11(13.10)	23(6.41)	0.0382	4.30	67(7.57)	34(7.52)	34(7.63)	0.9499	< 0.01
	3–5	409(92.33)	73(86.90)	336(93.59)			823(92.43)	414(92.48)	409(92.37)		
Cost composition	medical insurance	348(78.56)	68(80.95)	280(77.99)	0.5521	0.35	691(77.60)	343(76.73)	347(78.48)	0.5313	0.02
	own expenses	95(21.44)	16(19.05)	79(22.01)			199(22.40)	104(23.27)	95(21.52)		
Length of stay	4–9	111(25.06)	14(16.67)	97(27.02)	0.0511	1.95	222(24.94)	111(24.73)	111(25.15)	0.9062	< 0.01
	10–15	298(67.27)	62(73.81)	236(65.74)			607(68.16)	309(69.09)	297(67.22)		0.02
	16–21	34(7.67)	8(9.52)	26(7.24)			61(6.90)	28(6.18)	34(7.63)		0.01

Categorical variables are presented as n (%) ,PSW- propensity score weighted; BMI- Body Mass Index

Among all complications, pulmonary and urinary tract infection were the major ones in patients with acute ischemic stroke. The incidences of pulmonary and urinary tract infection in the orderly caregiving group were 20.24% and 21.43%, respectively, while those in the family caregiving group were 18.66% and 16.71%.The incidence of pressure ulcers in the family caregiving group was 2.51%, the incidence of falls in the family care group was 1.67%, whereas no fall cases were reported in the orderly caregiving group. Regarding the incidence of diarrhea, it was 9.52% in the orderly caregiving group and 9.75% in the family care group.

There was no significant difference between the two groups in terms of total hospitalization complications and nosocomial infections, including pneumonia, urinary tract infection, and diarrhea. The univariate analysis, as well as multivariate and PSW logistic regression analyses incorporating covariates including gender, age, BMI, comorbidities, smoking history, excessive drinking, and feeding methods and so on showed that the risk of pressure ulcer in the family caregiving group was low, OR value was 0.24 (95% Cl: 0.09–0.65, *P* = 0.0050), 0.28 (95% Cl: 0.10–0.78, *P* = 0.0151) and 0.31 (95% Cl: 0.16–0.61, *P* = 0.0006), respectively, with statistical significances. The family caregiving, therefore, was a protective factor for pressure ulcer (Table 2).

**Table 2:** Risk of complications of patients in different groups

Item	fullCohorts	Orderly	Family	Single factor OR (95%Cl)	Single factor *P* value	Multi factor OR (95%Cl)	Multi factor *P* value	OR (95%Cl) after PSW	*P* value after PSW
Pneumonia	84(18.96)	17(20.24)	67(18.66)	0.90(0.50–1.64)	0.7403	1.18(0.62–2.25)	0.6165	1.26(0.89–1.78)	0.1930
Urinary tract infection	78(17.61)	18(21.43)	60(16.71)	0.74(0.41–1.33)	0.3082	0.82(0.44–1.52)	0.5294	0.91(0.65–1.29)	0.6040
Diarrhea	43(9.71)	8(9.52)	35(9.75)	1.03(0.46–2.30)	0.9502	1.46(0.60–3.53)	0.4019	1.33(0.83–2.14)	0.2340
Falls	6(1.35)	0(0.00)	6(1.67)	-	0.9611	-	0.9619	-	0.9250
Pressure ulcer	17(3.84)	8(9.52)	9(2.51)	0.24(0.09–0.65)	0.0050*	0.28(0.10–0.78)	0.0151*	0.31(0.16–0.61)	0.0006*

*Significant at *P* < 0.05, PSW- propensity score weighted, Categorical variables are presented as n (%)

There was no significant difference in NIHSS score and lower limb muscle strength between the two groups before discharge. The proportion of patients with muscle strength above grade 3 in the family caregiving group was 90.60%, while that in the orderly caregiving group was 82.26%, reflecting the advantages of the former in upper limb strength (Table 3).

**Table 3:** Differences in functional rehabilitation of patients in different groups

Item	Classification	Full Cohorts	Orderly	Family	*P* value	Statistic
NIHSS score (before discharge)	0–5	776(87.16)	384(85.83)	392(88.52)	0.2307	1.44
	6–15	114(12.84)	63(14.17)	51(11.48)		
Barthel score (before discharge)	≤ 40	117(13.15)	69(15.43)	48(10.84)	0.0420*	2.03
	41 ~ 60	94(10.52)	48(10.70)	46(10.34)		
	>60	679(76.33)	331(73.87)	349(78.82)		
Muscle strength of affected upper limb (before discharged)	0–2	121(13.60)	79(17.74)	42(9.40)	0.0003*	13.19
	3–5	769(86.40)	368(82.26)	401(90.60)		
Muscle strength of affected lower limb (before discharged)	0–2	41(4.55)	22(4.90)	19(4.20)	0.6199	0.25
	3–5	849(95.45)	426(95.10)	424(95.80)		

*Significant at *P* < 0.05, Categorical variables are presented as n (%)

The hospitalization expenses of the family caregiving group were (19572.15 ± 11905.34) yuan, and the average daily hospitalization expenses were (1754.05 ± 1306.79) yuan; the hospitalization expenses of the orderly caregiving group were (17511.85 ± 13480.85) yuan, and the average daily hospitalization expenses were (1625.77 ± 1230.05) yuan. The those of the family caregiving group were significantly higher than those of the orderly caregiving group (P=0.0122 and 0.0004, respectively). The cost of orderly was charged at 220 yuan/day, leading to extra expenses in the orderly caregiving group of (2577.25 ± 1550.73) yuan accounting for about 15% of the total hospitalization expenses (Table 4).

**Table 4:** Difference of hospitalization expenses of patients in different groups unit: day and yuan

Item	Full Cohorts	Orderly	Family	*P* value	statistic
Hospitalization expenses	18535.94 ± 12290.80	17511.85 ± 13480.85	19572.15 ± 11905.34	0.0122*	-2.52
Average daily hospitalization expenses	1625.77 ± 1230.05	1498.98 ± 719.18	1754.05 ± 1306.79	0.0004*	-3.60
Expenses for orderly	1296.21 ± 1948.07	2577.25 ± 1550.73	0.00 ± 0.00	< 0.0001*	35.16
Proportion of orderly expenses	0.08 ± 0.11	0.15 ± 0.07	0.00 ± 0.00	< 0.0001*	48.52

*Significant at *P* < 0.05, Continuous variables with normal distribution are reported as mean ± SD

## Discussion

Stroke patients often lose their abilities of independent moving, activities of daily living, swallowing, and speaking, whose mood swings and non-cooperating with treatment and rehabilitation affect the medical and nursing work [12]. Experienced orderly can deal with various emergencies, allowing nurses to efficiently complete more professional work [13]. Patient’s family can truly understand the patient’s living habits and individual behaviors, detect abnormal physical changes in time, facilitate doctor-patient communication. In the care of hemiplegic patients, they can give more help to patients, such as passively move their limbs and encourage patients to do exercises independently. However, there are also limitations. For example, the quality of care provided by patients’ family may vary due to different family relationships; family may not fully compliant with hospital management; and they may urge patients to do more or harder exercises, due to impatient for the patient’s recovery.

Complications of stroke patients during hospitalization include infection, pressure ulcers, falls, thromboembolism, and seizures. The present study found that the incidence of pulmonary in all patients was 18.96%, which was lower than the range of 30%–65% reported in previous studies [14], and no statistically significant difference was observed in the infection incidence between the groups. This finding may be attributed to the well-defined inclusion and exclusion criteria adopted in the study, which excluded critically ill patients and high-risk populations. Furthermore, the balanced distribution of core baseline characteristics such as sex, age and comorbidities reduced the differences in infection rates caused by heterogeneity of the study participants.In this study, the incidence of falls was 1.35%. Since rehabilitation training for acute-phase patients was mainly conducted at bedside, there was no significant difference in the incidence of falls between the two groups either.There were 48% of falls that occurred in the first month after the most recent stroke [15]. Stroke patients who are close to having independent mobility have a higher risk of falling. In clinical practice, the falls usually occurred due to family’s encouragement or the orderly’s negligence. In this study, patients in the acute phase were mainly received bedside rehabilitation, and there were no significant difference in falls between groups. The high-intensity after stroke developing rehabilitation was associated with a significantly lowering of risk for pressure ulcers [16]. In this study, the incidence of pressure ulcers in the family caregiving group was lower than that in the orderly caregiving group (*P* = 0.042), which may be related to the family actively helping the patient to change positions, move the limbs, and encouraging the patient to exercise actively. Other findings showed that the muscle strength of affected upper limb before discharge were better in the family caregiving group than those in the orderly caregiving group, suggesting that the family were more active and effective in early rehabilitation than orderly. From the perspective of expenses, the total hospitalization expenses and average daily hospitalization expenses in the family caregiving group were higher than those in the orderly caregiving group. In clinical work, family attached close attention to medical work, including drug selection and nursing quality. On the other hand, the orderly caregiving group needs to extra expenses which were determined by the length of hospital stay. The expenses on orderly in China have not been included in the medical insurance system, which are borne by the patients’ side.

Family caregiving in this study was predominantly provided by spouses and adult children, while the involvement of other social contacts, including long-term friends and distant relatives was minimal. However, these relational dynamics also exerted potential impacts on patients’ rehabilitation. Studies have shown that, compared with caregivers who are other types of relatives, spousal caregivers tend to perceive a stronger sense of responsibility toward caregiving tasks and can provide patients with more emotional support [17]. Yet, they may lack professional caregiving knowledge and have a relatively poor understanding of rehabilitation-related concepts. Adult children, by contrast, demonstrate higher receptiveness to and better implementation of rehabilitation knowledge and complication prevention measures, but they often face the dual pressures of work and their own family obligations, which may lead to emotional outbursts toward patients and thus undermine the latter’s psychological rehabilitation.

Stroke rehabilitation is a long-term process, and its sequelae include lateral motor or sensory disturbances, spasticity, pain, depression, and even dementia [18], which seriously affect their abilities of daily living. After being discharged from the hospital, adapting to home life at beginning, and adapting to community life later on, the patients need to be accompanied. Family play an important role in this process, including falls prevention, drug use, nutritional support, psychological counseling, etc [19]. Many guidelines in and out of China suggested that stroke rehabilitation should be developed towards a family-centered care model [20]. However, long-term companionship will inevitably increase the burden of caregivers, affecting their quality of life and health. Caregivers may sacrifice family activities, leisure and social time, work and caring children [21]. Researches have found that the relationship between caregivers and care recipients may also change in the course of chronic disease, and this change may be reversed [22]. Orderly can minimize the potential damages to family functions [23], but the cost of care workers was also a burden to ordinary families.

This study only evaluated the effect of rehabilitation on acute stroke, and the following home care phase equally emphasized the importance of accompanying care. The long-term advantages of orderly and family need further research. We also put forward some suggestions on the improvement. First, the orderly management need to be improved. The training of orderly should be focused on professional skills, humanistic care, and personal protection. The incomes of the orderly and the management company can be more properly balanced. To protect the orderly’ rights and interests, it is recommended to purchase social insurance and increase their vacation time, etc., to improve their initiatives. By learning from the management experience of other countries, it is suggested to build a more standardized training system. Second, the guidance of care at home should be strengthened, such as providing health education to patients and caregivers before discharge, instructing patients to monitor blood pressure, blood sugar, and blood lipids, conducting video education on rehabilitation training, and paying attention to telephone or Internet follow-up. It is also needed to regularly assess patients’ motor and sensory functions, and encourage patients to carry out appropriate physical exercise or Traditional Chinese Medicine exercise. Furthermore, with the research and development of rehabilitation projects, wearable sensors can track changes in limb movements and transmit data. Portable rehabilitation therapy divices can assist patients in exercising and promote functional recovery.

## Conclusion

Family caregiving is a protective factor for pressure ulcer in ischemic stroke patients during hospitalization. The evaluation results of daily living ability and upper limb muscle strength of patients in the family caregiving group were better than those in the orderly caregiving group. Given the unique advantages of family caregiving, hospitals can improve the supporting policies. By strengthening the dissemination of professional knowledge and provide continuous and standardized care guidance for family caregivers. Orderly caregiving is a demand imposed on the medical service industry against the backdrop of an aging and low - fertility society. It is necessary to introduce a competition mechanism and promote the improvement of service quality through assessments, satisfaction surveys and other means.

## Data Availability

The datasets analyzed in the present study are available from the corresponding author on reasonable request.
